# Young Children and the Creation of a Digital Identity on Social Networking Sites: Scoping Review

**DOI:** 10.2196/54414

**Published:** 2024-02-21

**Authors:** Valeska Berg, Diana Arabiat, Evalotte Morelius, Lisa Kervin, Maggie Zgambo, Suzanne Robinson, Mark Jenkins, Lisa Whitehead

**Affiliations:** 1 School of Nursing & Midwifery Edith Cowan University Joondalup Australia; 2 Australian Research Council Centre of Excellence for the Digital Child Brisbane Australia; 3 Faculty of Nursing University of Jordan Amman Jordan; 4 Department of Health, Medicine and Caring Sciences Linköping University Linköping Sweden; 5 School of Education University of Wollongong Wollongong Australia; 6 The Centre for Evidence Informed Nursing, Midwifery and Healthcare Practice: A JBI Affiliated Group Joondalup Australia

**Keywords:** digital identity, children, social networking sites, sharenting, scoping review, perspectives

## Abstract

**Background:**

There is limited understanding of the concept of the digital identity of young children created through engagement on social networking sites.

**Objective:**

The objective of this scoping review was to identify key characteristics of the concept of digital identity for children from conception to the age of 8 years on social networking sites.

**Methods:**

This scoping review was conducted using the PRISMA-ScR (Preferred Reporting Items for Systematic Reviews and Meta-Analyses extension for Scoping Reviews) guidelines. The key databases searched were EBSCO, Web of Science, ProQuest ERIC, and Scopus. Gray literature sources (National Grey Literature Collection, ProQuest Dissertations and Theses, and Google Scholar) were also searched to identify unpublished studies. Articles were selected if they were published in English and reported data on the digital identity of children in relation to social networking sites.

**Results:**

The key terms used in the literature were *sharenting*, followed by *digital footprints* and *children’s identities*. Our study revealed 2 approaches to the creation of digital identity: *social digital identity* and *performative digital identity*. The articles in this review most commonly used the term *sharenting* to describe the behavior parents engage in to create digital identities for children on social networking sites. Motivations to post information about children differed among parents; however, the most common reasons were to share with friends and family and create digital archives of childhood photos, termed *social digital identity*. The second motivation was categorized as performative digital identity. The risk of digital kidnapping and identity theft associated with the creation of digital identities also influenced parents’ behaviors.

**Conclusions:**

The creation of a digital identity for children is an emerging concept. Our review develops a deeper understanding of sharenting behaviors that can be used to better support parents and their children in creating a digital identity with children and awareness of the potential future impact. We recommend that future studies explore the perspectives of children as key stakeholders in the creation of their digital identity.

## Introduction

### Background

Every post made on social networking sites contributes to the development of a digital identity. For some, this occurs naturally through their engagement with social networking sites, and for others, the process is planned or curated. Children and vulnerable populations can be represented on social networking sites without control over the creation of the digital identity developed on their behalf [1-7]. Children’s digital identities are often created before the child is born [8,9]. The creation of a child’s digital identity can start with parents sharing information about their soon-to-be-born or newly born child on social networking sites [3,10-12]. Digital identity development continues beyond the initial post as images, events, and milestones are shared with or without the permission of the child.

One of the major limitations of the literature on children and social networking sites is the underrepresentation of the voice of the younger child. There is little information available on social networking sites and their use and impact on children and even less from the perspective of children [13-16]. The lack of research with children is mainly attributed to the minimum age requirement for a child to register an account. Each social media site and app has its own criteria for minimum age requirements, which range from 13 to 16 years (13 with parental consent). It is common for parents to either post on behalf of their children or post (knowingly or unknowingly to the child) about their children between conception and the age of 8 years [17].

Although literature on the digital identity of children is emerging [8,12,18,19], evidence on the digital identities of adults has grown rapidly over the past 2 decades [20-25]. Despite the increase in the literature that explores adults’ digital identity, the key concepts related to processes and outcomes have not been established [1,20]. Approaches to define digital identity often draw on existing theories, such as the theory of self-presentation by Goffman [26,27]. Goffman [26] describes identity as performative and the world as a stage on which the act is taking place. The performance cannot take place without an audience who is there to validate the social performance [26]. Social networking sites are often seen as a stage in which one is actively trying to manage their impression or performance to be liked by others [28].

Research on adolescents’ digital identity (development) also draws on the theory by Goffman [26] and identity development theories such as the stages of psychosocial development were developed by Erikson [29], the identity status theory by Marcia [30], and the concept of networked publics by Boyd [31]. Identity development theories describe the adolescent years as the most important phase of identity development, and little is theorized about young children’s identity development [20,29,32]. However, Schachter and Ventura [33] argue that identity formation starts before adolescence and that parents play an active role in their children’s identity formation and later identity development. This aligns with the early formation of “digital” identities, which often starts with parents posting about their children on social networking sites.

### Objectives

There is limited understanding of the concept of digital identity for young children [21,34]. The purpose of this scoping review was to explore key characteristics in the literature on the concept of digital identity for children from conception to the age of 8 years on social networking sites. The review question was as follows: “What are the key concepts, definitions, and characteristics related to the concept of digital identity as generated through engagement with social networking sites for children from conception to the age of 8 years?”

## Methods

### Overview

A preliminary search of the Cochrane Database of Systematic Reviews and *JBI Evidence Synthesis* was conducted, and no current systematic or scoping reviews on the topic were identified. The updated methodological guidance for conducting a Joanna Briggs Institute scoping review was used in tandem with the PRISMA-ScR (Preferred Reporting Items for Systematic Reviews and Meta-Analyses extension for Scoping Reviews) to guide this review [35]. The completed PRISMA-ScR checklist can be found in Multimedia Appendix 1. A scoping review was assessed as the most appropriate method, where the purpose of this review was to identify and clarify concepts [36] regarding the digital identity of children. The scoping review protocol was registered with the Open Science Framework and can be retrieved via the web (see the reference for a link to the protocol) [37].

### Search Strategy

Relevant databases were searched using a constructed Boolean strategy with subject headings and keywords to reflect the inclusion criteria (the search strategy can be found in Multimedia Appendix 2). The first search was conducted between July 2022 and September 2022, and the second search was conducted between February 2023 and April 2023. The strategy was developed in conjunction with a specialist librarian. The search strategy, including all identified keywords and index terms, was adapted for each included database or information source. The databases EBSCO, Web of Science, ProQuest ERIC, and Scopus were searched. The reference lists of the included studies were cross-checked with search outcomes to identify studies not previously identified. Gray literature sources such as the National Grey Literature Collection, ProQuest Dissertations and Theses, and Google Scholar (the first 200 results) were also searched to identify unpublished studies.

The search terms were as follows: *child* OR *children* OR *infant* OR *toddler* OR *preschooler* (population) AND (*digital* AND *identity*) OR *“digital identity”* OR (*online* AND *profile*) OR *“online profile”* OR (*social* AND *presence*) OR *“social presence”* OR *sharenting* (concept) AND *social media* OR *Facebook* OR *Instagram* OR *Twitter* OR *Snapchat* OR *Tumblr* OR *“social networking”* (context).

### Inclusion and Exclusion Criteria

#### Overview

Studies of any research design that included the presentation of findings on digital identity in relation to children from conception to the age of 8 years on social networking sites were included if a full text could be retrieved. The viewpoint within the studies could be of the young person, family, health professionals, peers, and others. Further inclusion criteria were articles that were peer reviewed, written in English, and published between January 2000 and April 2023 inclusive. Gray literature was included if research findings were reported. No restrictions on the inclusion of studies were applied in relation to the geographic location or setting of the studies except for the generation of the data on social networking sites.

#### Participants

Social media related to children from conception to the age of 8 years was included. Data related to family members who posted about their children were also included.

#### Concept

The concept explored was digital identity on social networking sites in relation to children from conception to the age of 8 years. This review focused on web presence on social networking sites, and therefore, literature on digital identity that was purely data generated was excluded. Data-generated identities include, for example, log-ins, personal information saved on websites for identification purposes, and data saved while using apps and playing games. This type of digital identity is discussed elsewhere [38].

#### Types of Sources

This scoping review included both qualitative and quantitative studies. Quantitative study designs including experimental and quasi-experimental study designs, randomized controlled trials, nonrandomized controlled trials, before-and-after studies, interrupted time-series studies, analytical observational studies (prospective and retrospective cohort studies), case-control studies, and analytical cross-sectional studies were considered for inclusion. This review also considered descriptive observational study designs including case series, individual case reports, netnography, and descriptive cross-sectional studies for inclusion.

### Screening

Following the search, all identified references were imported into EndNote (version 20.1; Clarivate Analytics) for the identification and removal of duplicates and then exported to the Joanna Briggs Institute System for the Unified Management, Assessment, and Review of Information (Ovid) for a second identification of duplicates and the independent screening of titles and abstracts against the inclusion criteria by 2 reviewers [39]. Any differences between the reviewers regarding the inclusion or exclusion of articles for full-text review were discussed, and if not resolved, they were referred to a third reviewer. The full texts of the retained articles were independently assessed by 2 reviewers. Any differences between the reviewers were discussed and, if not resolved, they were referred to a third reviewer. The reasons for excluding studies at the full-text review stage were recorded. The study selection, screening, and reasons for exclusion at the full-text review stage are reported in the PRISMA (Preferred Reporting Items for Systematic Reviews and Meta-Analyses) diagram [35] in Multimedia Appendix 1.

### Charting the Data

Data extraction tables were developed with the team and used to ensure a uniform data extraction process. Data extraction was undertaken by a minimum of 2 reviewers. The selected studies were analyzed to identify the key characteristics, such as study design, aim, country of study, setting and context, participant characteristics (the age and gender of the children and their families), and sample size. Key terms and concepts related to children’s digital identity were identified, and themes and trends were charted. Where required and possible, the authors of the papers were contacted to request missing or additional data for clarification.

### Analysis and Presentation of Results

All articles in this scoping review were searched for key terms used in relation to the concept of digital identity. If the term was mentioned ≥2 times, it was included in the count. Key terms were included if they appeared in the main text, titles, abstracts, or keywords but not in references, footnotes, or headers.

Where variations of the term existed, all variations were analyzed as related to the core term. For example, for the core term *children’s identities*, variations such as *children’s identity*, *child’s identity*, *the identity of the child*, or *their (children’s) identity* were included. Similarly, variations of *sharenting* such as *oversharing*, *anti-sharenting*, and *grand-sharenting* [40] were analyzed as related to the core term *sharenting*.

The search was carried out using the PDF reader Nitro (Nitro Software, Inc), and words were copied and pasted into the search bar to avoid spelling mistakes. The search strategy included terms such as *identit* to quickly identify all terms related to identity, such as *online identity*, *digital identity*, and *social identity* (*identity* on its own was not counted).

Data were presented in tabular form, which allows for easy comparison between articles. A graphic was chosen as a way to demonstrate the relationships between key terms. Quantitative and qualitative data were extracted into tables to compare the studies, and qualitative data were sorted into key themes. Key trends are discussed in the Results and Discussion sections.

## Results

### Overview of Results

The search produced a total of 2573 abstracts, 1764 references from database and register searches, and 809 references from searches using other methods (refer to Multimedia Appendix 1 for the PRISMA flowchart [40]). Of the 1764 references, 652 (36.96%) were identified as duplicates, leaving 1112 (63.04%) references. There were no duplicates in the 809 references from other search methods. After title and abstract reviews were completed on all remaining references, 93.53% (1040/1112) of the articles were excluded from the database references and 99% (801/809) were excluded from the references from other search methods. This left 72 articles, of which 1 (1%) was excluded as there was no way to retrieve the full text and there were no contact details for the corresponding author [41]. Of the remaining 71 articles, after the full-text review, 50 (70%) were excluded, with the most common reasons being ineligible phenomena of interest (n=20, 40%), age (n=14, 28%), and the article not being about the child or children (n=8, 16%). This resulted in 21 articles. An additional hand search in March 2023 and April 2023 identified 7 articles for full-text review, of which 6 (86%) were included and 1 (14%) was excluded as it was not about the child or children. This resulted in a total of 27 articles included in this scoping review [7,9,10,17-19,40,42-61].

### Characteristics of the Studies

#### Participants

##### Overview

The total reported number of participants in this scoping review was 8643, comprising mothers (n=1768), fathers (n=585), grandparents (n=1), and participants reported collectively as parents (n=1841). In total, 4% (1/27) of the articles reported data from child participants (n=68) [59]. The remaining 4263 participants were not identified further. Overall, more female participants (n=4158) than male participants (n=1753) were reported in the articles.

The sample size of the included studies ranged from 1 [18] to 3472 [57] participants. Notably, 30% (8/27) of the articles did not provide sample characteristics [7,43-45,47,48,52,54]. This was due to the study context (eg, content analyses of social networking site posts and photos) [7,43-45,47,48,52,54] and the nature of the articles, such as books or reviews [54] (Table 1).

**Table 1:** Characteristics of the included studies.

Study, year	Study aim	Study design	Country	Setting and context	Identity type	Participants	Age	Sex
Ammari et al [19], 2015	To investigate how parents decide what to disclose about their children on SNSs^a^	Qualitative	United States	Sharenting and the shared responsibility of parents in managing their children’s online identities	SDI^b^	102 parents	Data unavailable	Male and female
Bare [43], 2020	To provide an overview of the images of children being posted to Instagram by parents under the hashtag #letthembelittle	Qualitative	United States	Content analysis of Instagram posts of children with the hashtag #letthembelittle.	SDI and PDI^c^	Unspecified	Data unavailable	Data unavailable
Benevento [44], 2022	To understand how photographs shared on social media connect and express values regarding childhood	Narrative inquiry	Not specified	Analyzing Instagram postings and comments on photos of children on 2 hashtags—#letthekids and #fashionkids	SDI	Not specified	Data unavailable	Data unavailable
Bezakova et al [45], 2021	To identify the extent of the problem of sharing content on minors with family members on social media (s*harenting*), identify legal solutions to the problem, and point out the importance of adequate social mechanisms (media and marketing) to raise awareness of the issue	Analytical-synthetic and comparative research methods	Not specified	Analyzing sharenting of sensitive data on social media, comments, reviews, blogs, web portals, and emails. Identifying legal solutions to protect children.	SDI	Not specified	Data unavailable	Data unavailable
Briazu et al [46], 2021	To investigate how the risks and benefits alongside psychosocial variables affected the Facebook sharenting behavior of mothers of young children	Mixed methods	United Kingdom	Facebook sharenting behaviors of mothers	SDI	190 mothers of young children	62.6% were aged between 25 and 34 y	Female
Brosch [10], 2016	To learn about parents’ habits regarding their children on Facebook, especially how much and what kind of information about their children they share	Social media ethnography	Poland	Sharenting on Facebook. Exponential nondiscriminative snowball recruiting.	SDI	168 parents with a child or children aged <8 y	Data unavailable	Data unavailable
Choi and Lewallen [47], 2018	To examine how children are represented on Instagram and how children are depicted in relation to traditional stereotypes	Mixed methods	United States	Content analysis of 510 photos of children on Instagram on children’s gender and racial representations on social media	SDI and PDI	Not specified	Data unavailable	Data unavailable
Cino and Dalledonne Vandini [40], 2020	To investigate how boundaries of children’s social media presence are understood and experienced within interacting systems regarding the relationship between MILs^d^ and DILs^e^	Literature review and qualitative study	United States	Digital dilemmas on their children’s digital footprints, privacy, and social media presence created by members external to the family, such as the child’s teacher. Analysis of parents’ posts on a BabyCenter community, a web-based parenting forum.	SDI	300 parents	Most were female. Specific data are unavailable.	Data unavailable
Dobson and Jay [18], 2020	This paper explored the representation of children and family life, with an emphasis on the “image of the child” that exists on Instagram.	Qualitative	Australia	Perspectives and experiences of an influencer parent sharenting photos on Instagram	SDI and PDI	1 mother	Data unavailable	Female
Er et al [48], 2022	To investigate *sharenting* during the early COVID-19 pandemic and quarantine periods	Qualitative	Turkey	Sharenting during the pandemic and quarantine period. Descriptive content analysis of the Instagram profiles of the parents—401 posts from Instagram	SDI	Unspecified	Data unavailable	Data unavailable
Fox et al [50], 2022	To explore first-time fathers’ vulnerabilities and decisions to engage in sharenting, especially given that marketers seek to connect with new parents on social media via engagement tactics that prompt sharenting	Mixed methods	United States	First-time fathers’ willingness to sharent on social media and their level of perceived sensitivity to their children’s information. Web-based survey on Amazon Mechanical Turk using Prime Panels and grounded theory.	SDI	75 first-time fathers	Aged 20 to 40 y	Male
Fox and Hoy [49], 2019	Study 1: to explore mothers’ expressions of vulnerability and how these relations can be linked to their motivations for sharing children’s PII^f^ on social media. Study 2: to explore mothers of young children in a Twitter chat and the extent to which they post children’s PII, as well as the mother’s vulnerability.	Mixed methods	United States	Qualitative: interaction of consumer vulnerability of the mother and the reasons and decision to post about their children on social media. Quantitative: interaction of a brand—Carter’s, Inc and Children Apparel—with the engagement of mothers on Twitter.	SDI	Study 1: 15 mothers; study 2: 122 participants	Study 1: aged 24-40 y; study 2: data unavailable	Study 1: female; study 2: data unavailable
Hashim et al [51], 2021	To investigate the trends, motives, or purposes behind sharenting by Malaysian parents and their awareness (or lack thereof) of its related privacy issues	Qualitative	Malaysia	Mothers’ motives to sharent and the type of content they post frequently and like to update their status with or post on social media	SDI	40 mothers	52.5% were aged between 31 and 40 y	Data unavailable
Holiday et al [52], 2022	To identify how parents self-present in their sharenting posts	Qualitative	United States	Self-representation on Instagram posts about their children	SDI and PDI	Unspecified	Data unavailable	Data unavailable
Jorge et al [17], 2022	To explore how Cristiano Ronaldo, his partner, and his mother shared information about his children on Instagram between 2018 and 2020	Qualitative	Portugal	Sharenting of a celebrity, Cristiano Ronaldo, and his family members. The digital identity of Cristiano Ronaldo’s children analyzed through sharenting by Ronaldo, his partner, and his mother on Instagram.	SDI and PDI	3 participants (mother, father, and grandmother)	Data unavailable	Data unavailable
Kopecky et al [53], 2020	To investigate the type of content that parents publish about their children and compare this behavior between Czech and Spanish parents	Quantitative study	Czech Republic and Spain	Comparing sharenting content, extent, and behaviors in 2 countries. The study was conducted on the web (Google Forms distributed through Facebook, Instagram, email, and WhatsApp channels)	SDI	1093 Czech parents and 367 Spanish parents	Czech parents aged 25 to 64 y; Spanish parents aged 21 to 61 y	Men and women
Kumar and Schoenebeck [9], 2015	To gather mothers’ narratives and experiences about sharing baby photos on Facebook. To show how identity performance allows mothers to enact—and receive validation of—good mothering.	Qualitative study	United States	Attitudes, opinions, and experiences of sharing baby photos on Facebook and mothers’ perceptions of Facebook and other sites	SDI	22 mothers	Aged 25 to 39 y	Female
Kumar [54], 2021	To investigate how power works through 3 fields of discourse that govern parents’ social media conduct	Review and qualitative study—“thinking with theory” method	United States	Governmentality and parents’ conduct in sharenting	SDI	Unspecified	Data unavailable	Data unavailable
Latipah et al [55], 2020	To describe the sharenting model by millennial parents as a process of exchanging information between parents in parenting, mentoring, education, and child development	Phenomenological approach	Indonesia	Motives, impact, and ways of sharenting. Interview was completed via the web.	SDI and PDI	10 parents	Aged 24 to 35 y	5 female and 5 male
Leaver [7], 2020	To investigate how exactly the digital communication and sharing of and by parents about their children can be balanced with children’s rights to privacy both in the present and, more challengingly, in the future	Critical review of parenting practices through examples	Australia	Sharenting children’s sensitive information on Instagram, Facebook, wearables, and apps (Owlet Smart Sock and Peakaboo Moments); web safety; and children’s rights to opt out	SDI and PDI	Unspecified	Data unavailable	Data unavailable
Marasli et al [56], 2016	To investigate the use frequency and the content of social media sharing and investigate the information a group of parents shared on the web about their children via content analysis	Mixed methods	Turkey	Sharenting on Facebook	SDI	219 parents	41.7% were aged 31 to 40 y	Data unavailable
Mascheroni et al [62], 2023	To investigate the patterns of sharing among a nationally representative sample of parents of children aged 0 to 8 y. To identify the presence of recurrent sharenting styles. To examine the relationship between sharenting styles and parents’ sociodemographic information and between sharenting styles and parental practices of privacy management adopted to govern their children’s social media presence.	Quantitative	Italy	Sharenting styles, extent of sharenting, and parents’ privacy management practices	SDI	1000 Italian parents	Aged 18 to 54 y	Male and female
Minkus et al [57], 2015	To measure adults’ sharing of children’s PII in web-based social networks, namely, Facebook and Instagram	Mixed methods	United States	Analysis of images shared on Facebook and Instagram	SDI	2383 Facebook users and 1089 Instagram users	≥18 y	Women and men
Morris [58], 2014	To provide insights into the types of child-related content that mothers of infants and toddlers are willing to share on SNSs	Mixed methods	United States	How mothers of young children use Facebook and Twitter and mothers’ perceptions on the appropriate site on which to share photos of their children. Survey was completed on the web.	SDI	412 mothers	Aged 19 to 46 y	Female
Sarkadi et al [59], 2020	To investigate children’s thoughts about sharenting	Quantitative	Sweden	Children’s views on sharenting. Survey was completed on the web.	SDI	68 children	Aged 4 to 15 y	Two-thirds were boys, and one-third were girls
Turgut et al [60], 2021	To investigate what factors affect what parents share on social media about their children	Qualitative study	Turkey	Sharenting and its associated factors and parents’ views on legal liability	SDI	88 parents	Aged 22 to 45 y	Data unavailable
Wagner and Gasche [61], 2018	To investigate what factors parents consider when disclosing personal information about their children on SNSs and what strategies they apply	Qualitative	Germany and Austria	Parents’ thoughts on drivers and inhibitors of disclosing children’s photos on SNSs	SDI	220 mothers	Data unavailable (mean age 31.1 y)	Data unavailable

^a^SNS: social networking site.

^b^SDI: social digital identity.

^c^PDI: performative digital identity.

^d^MIL: mother-in-law.

^e^DIL: daughter-in-law.

^f^PII: personally identifiable information.

##### Study Origin

Of the 27 studies, 11 (41%) were conducted in the United States [9,19,40,43,47,49,50,52,57,58], 3 (11%) were conducted in Turkey [48,56,60], and 2 (7%) were conducted in Australia [2,18], followed by 1 (4%) study conducted in both the Czech Republic and Spain [52], 1 (4%) conducted in Germany and Austria [61], and 1 (4%) from each of the following countries: the United Kingdom [46], Malaysia [51], Poland [10], Sweden [59], Italy [62], Indonesia [55], and Portugal [17]. The remaining 7% (2/27) of the studies did not name the country of data origin [44,45].

##### Context

The main social networking sites used were Instagram and Facebook. A total of 26% (7/27) of the studies focused on Instagram [17,18,43,44,47,48,52], and 15% (4/27) of the studies focused on Facebook [9,10,46,56]. The remaining studies focused on social media more broadly.

#### Study Design

In total, 48% (13/27) of the studies used a qualitative approach [9,10,17-19,43,44,48,51,52,55,60,61]. A total of 26% (7/27) of the studies used a mixed methods approach [46,47,49,50,56-58]. In total, 11% (3/27) of the studies used a quantitative design [30,53,59]. A total of 7% (2/27) of the studies used both qualitative and literature review methodologies [40,54], and 4% (1/27) of the articles were book chapters [7].

### Key Terms and Concepts Used to Describe Digital Identity

In this first part of the *Results* section, we explore key terms and concepts used in relation to the concept of the digital identity of children on social networking sites. We then explore the concept of digital identity in relation to 2 types of behaviors that underpin the development of young children’s digital identity.

#### The Key Term Sharenting

##### Overview

The term *sharenting* was the most commonly used term in the literature (21/27, 78% of the articles) on the development of children’s digital identities [7,10,17,40,44-54,56,59,60]. Of the 27 studies, 5 (19%) studies discussed the term in more detail and provided a definition of *sharenting* [40,45,47,49,50]. Bezakova et al [45] explained the term *sharenting* as “the overuse of social media by parents or legal guardians who share photos or various home videos of minors with the virtual community,” whereas Brosch [10] defined *sharenting* as “the practice of a parent to regularly use the social media to communicate a lot of detailed information about their child” and drew on the Collins dictionary definition. All authors appeared to share a similar understanding of the term *sharenting*. Thus, the definition of *sharenting* is widely accepted and used frequently in the context of the digital identities of children on social networking sites.

##### Digital Footprint

A total of 48% (13/27) of the articles referred to the concept of digital footprint(s) [7,9,10,19,40,45,46,48,50,53,54,60,62]. The term *digital footprints* was sometimes used interchangeably with the term *digital identity*. It often came down to the authors’ preference for wording to describe the creation of digital identities for children. For example, Brosch [10] and Bezakova et al [45] explained that children’s digital footprints are mostly created by parents early in their child’s life, sometimes before or just after the birth of the child or during infancy [10,45]. Brosch [10] further explained that 10.7% of Polish parents in their sample created digital footprints for their unborn children by posting sonogram images, and 8.3% shared photos of the expectant mother on Facebook. As illustrated by this example, the term *digital footprints* was used synonymously with the term *digital identity*.

When the risks of sharing children’s content on the web were discussed, the term *digital footprints* was often chosen. Kumar and Schoenebeck [9] discussed the risk of mothers creating digital footprints for their children in relation to the benefits of receiving validation. Mothers in their study were hesitant and uncertain about how their photo-sharing behavior might affect their children’s online identity later and restricted their sharing to pictures that were cute and funny and showed milestones. Nevertheless, they found that the benefits of receiving validation via shared content outweighed the mothers’ concerns about digital footprints and oversharing. The authors introduced a new term, *privacy stewardship*, to describe “the responsibility mothers take on as they consider what kinds of baby photos are appropriate to share and the implications for their children’s digital footprint.” In line with this, Cino and Dalledonne Vandini [40] described the pressure and responsibilities of motherhood as mothers are eager to and expected to actively manage their children’s digital footprints. The literature suggests that the management of children’s digital footprints and identities is mostly considered to be the responsibility of parents, especially mothers [7,9,40,62].

#### The Use of the Term or Concept of Identity

The different types of identities that were mentioned in relation to children’s digital identities on social networking sites are discussed in the following sections.

##### Children’s Identities

The term *children’s identities* or variations of this term (eg, *child’s identity*) was used in 44% (12/27) of the articles [7,9,17,19,43,44,48,52-54,56]. The term *children’s identities* was used to represent a broad concept that often encompassed other subterms or concepts related to identity. A total of 26% (7/27) of the articles that included the term *children’s identities* further discussed the concept of *online identity* [9,17,19,43,45,53,60], and 15% (4/27) of the articles discussed the term *digital identity* [17,54,60,62].

##### Online Identity

All articles that used the term *online identities* discussed how parents were the creators of their children’s identities on the web [9,17,19,43,45,53,60]. Similar to the other concepts related to the digital identity of children, *online identity* could often be used interchangeably with the term *digital identity*. However, the context in which *online identity* was used differed from that in which the other terms were used. Of the 27 studies, 5 (19%) studies discussed children’s online identities in the context of children’s rights and agency over their online identity and the missing consent from children to allow their parents to post about them on the web [17,19,43,45,53].

##### Digital Identity

The literature did not generate an accepted definition of digital identity; however, some authors briefly discussed the concept and its relationship with *sharenting*. Kumar [54] linked the concepts of digital identity and sharenting: “sharenting is potent thanks to the concept of a ‘digital identity,’ also called a digital persona, profile, legacy, trail, footprint, or presence” and “Sharenting discourse portrays the creation of a digital identity as a choice, one best left to the child.”

Mascheroni et al [62] also linked the 2 terms by discussing the consequences of sharenting on children’s digital identity: “Generally speaking, almost half of the parents are reportedly aware of the consequences of sharenting for children’s digital identity, but regular sharers show a lower average value, suggesting a lower degree of awareness.”

Jorge et al [17] discussed the term *digital identity* in more detail by exploring how celebrity sharenting contributes to the construction of children’s digital identities. They found that the parents shared information and photos that aligned with the theme of happy and grateful parenthood and that the family posts represented the children as the extended selves of the father, stepmother, and grandmother.

Thus, there is an understanding that the digital identities are created by parents through sharenting. Here, sharenting is seen as the action (sharing information about the child), and the digital identity is described as the consequence or outcome of the sharenting behavior. Although sharenting was well defined, definitions for children’s digital identity were not provided in the articles.

Other terms or concepts that included the word *identity* were used less frequently; for example, *relational identity* was mentioned in 7% (2/27) of the articles, whereas the terms *identity performance*, *mediated identity*, *private identity*, *social identity*, *social media identity*, and *moral identities* only appeared each in 4% (1/27) of the articles. Overall, most articles (19/27, 70%) in this review discussed some form of identity in relation to children’s presence on social networking sites.

*Sharenting* is the behavior that parents engage in when sharing information about their children on social networking sites. This creates long-lasting *digital footprints* on the web that form children’s *digital identities*. The literature has identified a number of risks related to the creation of children’s digital identities on social networking sites, such as *digital kidnapping* and *identity theft*, especially if the information that was shared contained *personally identifiable information*. These areas will be explored in relation to the concept of the digital identity of young children.

#### Safety: Digital Kidnapping

A total of 11% (3/27) of the articles in this review discussed the concept of digital kidnapping [43,48,51]. The terms *identity theft*, *personally identifiable information*, and *privacy stewardship* were used in 7% (2/27) of the articles in this review [9,46,49-51,54]. The term *digital kidnapping* is defined as “people who steal a child’s identity and photo on social media and pass the child off as their own” [48]. Digital kidnapping is described as one of the risks of creating digital identities for children by sharing images, especially those that include personal information about the child and reveal the child’s face [43,48]. Hashim et al [51] found that Malaysian mothers were concerned about digital kidnapping and identity theft and, therefore, were conscious of not sharing locations in their posts and actively hid information regarding places and their children’s names and dates of birth.

#### Children’s Digital Identity as an Extension of Parents’ Digital Identities

A total of 7% (2/27) of the articles discussed the concept of extended self [17,52]. These 2 articles also discussed the term *relational identity*. In the article by Holiday et al [52], the authors discussed the theory of the “extended self” and applied it to the concept of sharenting. The authors described parents’ engagement in sharenting as fundamental to their identity as parents, which the authors argued says more about the parent as an individual than about the depicted child. Following this thought, sharenting is seen as a form of parents’ self-presentation that includes children as a component in the deﬁnition of the self.

Jorge et al [17] also described parents’ representation of children on social networking sites as the extended selves of family members. When children’s digital identities on social networking sites are interpreted as extensions of their parents’ or family members’ identities, parents’ and family members’ identities form part of the child’s digital identity. Accordingly, some articles in this review (4/27, 15%) discussed the digital identity of parents, mothers, and families in relation to the child’s digital identity [9,49,54,62].

Overall, the review of the key term and concepts related to digital identity shows that there is limited research defining key terms such as children’s *digital identity* and *digital footprints*, whereas *sharenting* is a commonly used and widely accepted term that is clearly defined.

### Content and Image Analyses

#### The Development of Social and Performative Digital Identities

The synthesis of the data generated through content and image analyses generated 2 types of digital identity: “social digital identity” and “performative digital identity.” Children’s social digital identity creation involves parents who create their children’s digital identity by sharing information such as everyday activities and milestones without links to commercial products or promotion of their children. Parents’ motivation to create social digital identities for their children is most often to share with family and friends and keep a digital diary [9,10,51,52,54,61], whereas children’s performative digital identity is created when parents promote or market their children, often for their own benefit, for example, to promote their clothes and brands [18,44,52]. This means that parents post information and photos of their children to convey a picture of the child that can deviate from the actual identity of the child. These posts often present the child in a neat and fashionable way and can include links to products that parents obtain a financial share of. For example, “mummy” or fashion bloggers (eg, #fashionkids) create performative digital identities for their children that mostly benefit them and often disregard the needs of the child [18,63].

#### The Use of Social and Performative Digital Identities in the Literature

##### Overview

Most articles (18/27, 67%) discussed social digital identities exclusively [9,10,19,40,42,45,46,48-51,53,54,56-58,60,61], whereas 30% (8/27) discussed performative digital identities [7,17,18,43,44,47,52,55]. Social digital identities were mostly created on Facebook or discussed in a social media context in general, whereas performative digital identities were mostly created on Instagram. A summary of the types of posted content is presented in Table 2. The percentages indicate the proportion of articles that discussed the different topics.

**Table 2:** Analysis of posted content related to children on social networking sites (N=27).

Content	Total articles, n (%)	Activity or leisure time, n (%)	Events (birthdays or family), n (%)	Posing or influencer or making income, n (%)	Developmental stages or milestones, n (%)	Family holidays or outings, n (%)	Embarrassing or cute, n (%)	Face visible, n (%)	Name or DOB^a^, n (%)	Nudity, n (%)
Social DI^b^	18 (67)	11 (61)	13 (72)	1 (6)	6 (33)	3 (17)	8 (44)	6 (33)	7 (39)	5 (28)
Performative DI	8 (30)	7 (88)	2 (25)	6 (75)	1 (12)	1 (12)	2 (25)	3 (38)	2 (25)	3 (38)

^a^DOB: date of birth.

^b^DI: digital identity.

Social digital identities were often created through images of events such as birthdays and family gatherings, whereas most of the studies that demonstrated a performative digital identity (8/27, 30%) included images and descriptions of children posing for photos, and in some cases, the family made an income from these posts [7,17,18,43,44,47,52,55].

In the following sections, we explain what information (including text and photos) parents typically share when creating social and performative digital identities for children and what motivates them to share this information.

##### Social Digital Identities

###### What Parents Share When Creating Social Digital Identities for Their Children

Most studies (10/27, 37%) reported that parents created social digital identities for their children by sharing their happy moments. Brosch [10] found that these happy moments were often recorded during daily life activities, outings, and special events (95.6%). Similarly, most of the mothers in the study by Briazu et al [46] shared information about special days (72.7%) or social activities (52.6%), and some shared information about health (6.7%) or educational issues (5.2%). Brosch [10] found that many parents revealed private information about their children by sharing posts containing images of their children’s birthday parties (23.2%), baby videos, birth certificates, kindergarten diplomas, or art (32.7%), as well as sonogram images (10.7%). Information about the child was also shared via posts containing information such as the child’s name and date of birth (48.2%). Brosch [10] also found that some of the posts contained embarrassing photos (eg, nude or seminude pictures of the child during bathing or at the beach), photos in which children were in distress (eg, crying or angry), or photos in which children were covered in food after dinner (eg, chocolate on their faces).

Kopecky et al [53] surveyed parents from the Czech Republic and Spain and found that these parents shared photos of celebrations, family moments, holidays, important milestones, and photos that parents considered to be cute or funny. Most parents reported sharing content in which the child could be identified (by face) but did not include sexual content (81.7%). One-ﬁfth of parents shared photos in which the child was partially exposed to the extent that the identity of the child could be determined. A small proportion (3.5%) of parents from the Czech Republic reported sharing nude photos of their young children.

Er et al [48] investigated sharenting behaviors at the beginning of the COVID-19 pandemic. They found that mothers posted more often than fathers and that most posts contained photos and some contained videos of the children. Of the 226 posts they analyzed, 207 included the children’s faces, with a limited number of parents blurring their children’s faces (n=17). In line with the other studies, the posts were generally happy, for example, expressing the joy of spending time with children and love toward children and showing how children and the family happily played games, cooked, or learned together. The daily lives of the children were also posted, including birthdays, vacations, and anniversaries. A smaller proportion of posts expressed unpleasant situations during the COVID-19 pandemic, such as boredom, complaints, and unhappiness with quarantine.

Cino and Dalledonne Vandini [40] explored the digital identities that are created for children by the mothers’ mothers-in-law and the conflict that this raises with the mothers. The content is either shared before the birth of the child (eg, pregnancy status of the mother, gender reveal, or labor) or afterward (eg, daily life activities) and usually against the will or knowledge of the mother.

Fox et al [50] investigated first-time fathers’ sharenting behavior and found that fathers tried to avoid posting sensitive information (eg, their naked child). However, they did post about everyday activities such as going to the park, playing, birthdays, and firsts (eg, first tooth). Fathers were aware of security risks and, therefore, hid their children’s faces and names.

Hashim et al [51] found that parents mostly shared social events (eg, vacations, events, family activities, and outings; 29.3%), moments (eg, good, funny, happy, important, or special moments; 25.3%), day-to-day activities (13.3%), memories of their children (12%), school activities (10.6%), food (4%), antics (2.6%), and milestones (2.6%) about their children.

Kumar and Schoenebeck [9] interviewed mothers about their sharenting experiences. Mothers described the photos that they shared about their children as cute and funny and explained that the photos often contained family or friends and developmental milestones of the children.

Marasli et al [56] found that the most common theme parents shared about on Facebook was special days (81.4%), such as birthdays, graduations, and year-end shows, followed by social activities (54.98%) and educational issues (30%). Less commonly shared themes included sports and arts activities (18.96%), play activities (17.54%), health issues (12.8%), and recommendations about products for children and informatics (12.32%). Most parents in this study (63.77%) also reported that they liked sharing pleasant things about their children.

Minkus et al [57] used a web-based application programming interface called Face++ to analyze Facebook and Instagram photos. The software identified children via age estimates based on the faces in the photos. Over 25% of the photos on Facebook and 16% of the photos on Instagram with children aged 0 to 7 years had comments that revealed the children’s names, and 2.7% (Facebook) and 5% (Instagram) included the word *birthday*. The authors were also able to infer the children’s last names from the parents’ last names. Overall, 5.6% of Facebook accounts and 19% of Instagram accounts with child photos revealed the name and date of birth of the children, which is enough information to identify them. By further linking the parents’ Facebook accounts with public records (eg, voter registration records), the authors were also able to identify the address of the parents and children.

###### Parents’ Motivation to Create Social Digital Identities for Their Children

In this section, we explore mothers’, fathers’, and mothers-in-law’s motivations for creating social digital identities for their children on social networking sites. Briazu et al [46] found that mothers’ motivations or perceived benefits of posting about their children were to build connections, gain practical benefits such as asking for parenting advice, gain emotional benefits (eg, pride and joy from their children), and help others, and some mothers did not identify any benefits.

Fox and Hoy [49] found that the desire to be a “good” mother motivated mothers’ sharenting behavior. Mothers used sharenting as a coping strategy. They shared their experiences as mothers and information about their children to seek affirmation and social support from others. The authors also explored mothers’ motivations *not* to post about their children. Mothers focused on portraying the “right” image of the child and avoided posts that potentially could have made them look like a “bad” parent. It was also important to mothers in this study that their children would not be upset or embarrassed by their posts later in life.

Kumar and Schoenebeck [9] found that most mothers in their study used Facebook as an archive for their children’s photos. It was important to these mothers to portray their children and themselves in a favorable light and to receive validation and support as mothers.

Wagner and Gasche [61] investigated German and Austrian mothers’ decision-making processes and strategies when sharing about their children. Most mothers indicated that the costs of sharing photos of their children on the web outweighed the benefits, and therefore, more than half of the mothers (60%) never shared photos of their children on social networking sites. The mothers’ main motivation to share was social participation (to inform others, to keep others up to date, and to document the children’s development), followed by showing how proud they are of their children and the need to be liked, approved of, and accepted by others.

Fox et al [50] found that fathers’ motivation to share was not to gain support from others but rather to express humor or spotlight themselves as fathers. Overall, fathers made fewer sharenting decisions, and the main responsibility of sharenting most often lay with the mothers [50].

Hashim et al [51] found that the most common motivation (42.8%) for Malaysian parents to share about their children was to save memories of them. Social networking sites served as an archive or journal for them to refer to at a later stage. The second most common motivator (31.6%) was the desire to share their experiences, information, activities, and feelings about raising children. Other motivations included being influenced by other social media users; staying connected and engaged with others; and motivating, encouraging, and inspiring other parents. In line with this, Turgut et al [60] described parents’ motivation to post about their children as related to keeping in touch with others (eg, relatives and friends) and recording and memorizing their children’s development. Brosch [10] found that the number of Facebook friends was a significant predictor of sharenting.

Cino and Dalledonne Vandini [40] investigated the motivation of mothers-in-law to post about their grandchildren. They reported that grandmothers’ motivation stemmed from a desire to show excitement for the grandchild, which was often at the cost of the parent’s desire for agency over their children’s digital identities. However, it was noted that grandparents might be less knowledgeable about the internet and web safety and are potentially naiver about sharing information about their grandchildren on the web.

##### Performative Digital Identities

###### What Parents Share When Creating Performative Digital Identities for Their Children

Posts that contribute to a child’s performative digital identity creation are usually well planned out to present the child in a fashionable or favorable way. Benevento [44] investigated posts with the *#letthekids* and *#fashionkids* hashtags. These are often used by parents who create performative digital identities for their children by sharing well-prepared posts that have been planned out. The hashtag *#letthekids* emerged as a counter to the more established hashtag *#fashionkids*; it stands for “let the kids dress themselves.” The author found that *#fashionkids* photos often show the child alone during structured activities outdoors. Children are often displayed smiling or with still expressions posing with their possessions (eg, clothing and accessories). The attention is drawn to the child and their outfit rather than the location or activity. The background locations include well-maintained spaces such as parks, backyards, and playgrounds as well as home settings (eg, bedrooms and kitchens). Although children are often presented as posing with a focus on their clothes, these are most often casual.

In contrast, *#letthekids* photos often show the child during unstructured activities, such as during play, eating in their home environment, or in nature (eg, forest). This hashtag often displays children acting on their own, for example, while playing with their toys in their room, but also sometimes includes family members. The children in the *#letthekids* hashtag often look away or are shown from behind, as if they are not aware of the photo being taken. Interestingly, *#letthekids* posters upload more professional photographs than *#fashionkids* posters and more naked or seminaked pictures of their children than *#fashionkids* posters [44].

Choi and Lewallen [47] investigated children’s gender representations on Instagram and found that parents posted more about their female children than about their male children and generally presented both their female and male children with positive emotions in white or gender-typical (ie, pink and blue) clothes. Children on Instagram were often displayed as playing or having fun in indoor settings by themselves. Girls were found to be frequently displayed as engaging in fashion.

Holiday et al [52] explored how parents self-presented in their children’s presentation on Instagram. The authors identified 3 presentational categories: *polished*, *promotional*, and *intimate*. Photos in the *polished* category displayed children as visually appealing and suggested that parents invested time and effort in the post to portray an idealized image of the child. The parents were presented as favorably themselves, with possessions including the child. The attention was often directed toward the parents, not the children (via the text or image). Children in this category served as accessories (eg, in the parents’ arms or on the side of the photo). Parents typically presented themselves as their “ideal self” in this category. The *promotion* category included posts in which parents used their children to promote their own skills, competencies, services, or products. Finally, the *intimate* category portrayed children more realistically without perfectioning of the image. With a strong focus on the child in the *intimate* category, more information is revealed about the child, which adds to the child’s digital identity [52].

Jorge et al [17] explored celebrities’ creation of their children’s digital identities through sharenting. The authors analyzed Cristiano Ronaldo’s family’s sharenting practices and the portrayal of the children as the parents’ extended selves. The results showed that celebrity sharenting contributes to digital identities through the themes of happy and grateful parenthood and the representation of children as the extended selves of the father, stepmother, and grandmother. Finally, Latipah et al [55] found that millennial parents shared content about their children related to everyday activities that are perceived as fun and that are often displayed as esthetically pleasing, with some posts including the promotion of products.

###### Parents’ Motivation and Motives for Creating Performative Digital Identities for Their Children

Parents who engage in performative digital identity creation for their children have several motives for sharenting. Some parents want to pass on knowledge and educate other parents by providing advice, products, and insights into their daily life activities [18,55], whereas others’ motive is to primarily promote their products or clothes [44,52]. In the *promotion* category in the study by Holiday et al [52], the motivation behind posting was often to promote products or services to other parents, whereas parents’ motivation in the *intimate* category was often to preserve memories, which is in line with our findings on the motivation to create social digital identities.

Dobson and Jay [18] found that the motive of their case study was to connect with others as the family lived in a rural area. The mother reported that she had made friendships on the web and that followers empathized with her posts and offered support and a sense of community.

In the study by Latipah et al [54], parents’ motivation to share about their children was to receive affirmation and social support and to demonstrate the ability to care for their children, social participation, and documentation.

The only study that included children as participants could not be classified as either “performative” or “social” digital identity. In this study, children were asked for their opinion on sharenting [58]. Children aged 4 to 15 years indicated that it is not OK for parents to post photos of their children (them) on social networking sites, whereas sending the photos to relatives was more accepted by the children in the study. The lowest (least acceptable) scores were found among the youngest children (aged 4-6 y) in the study. Irrespective of the participants’ age, children wanted to be asked before their parents took or shared photos of them, and they wanted their answers to be listened to.

## Discussion

### Summary of Principal Findings

#### Overview

This scoping review identified 27 studies. Participants included mothers and fathers (collectively reported as parents) and grandparents. On the basis of the analysis of the key terms and concepts used in the literature, the following description of how these relate to one another was developed. The creation of a child’s digital identity is developed through the behaviors of parents, most referred to as *sharenting*. The behavior of parents through the decisions on the web they make creates a digital identity that can be described as social digital identity or performative digital identity. We found that much of the literature on the concept of the digital identity of children reports on parents, especially mothers, and their sharenting behavior on social networking sites. The most used terms related to digital identity in the literature are *sharenting*, followed by *digital footprint* and *children’s identity*. The term *sharenting* is well defined and popular among researchers and the media, whereas the term *digital identity* was less commonly used. We found that the term *digital footprint* was more commonly used than *digital identity*; however, clear definitions were also lacking in the articles in this review. Common across all terms was parents making decisions about what to share about their children, mostly without the children’s consent.

The term *digital identity* is more commonly used in the literature on adults [20-25,64,65]. However, we expect a rise in the term digital identity in relation to children in the coming years as there has been a steep increase in research that focuses on the consequences and risks of sharenting [50,66,67]*.* The use of digital identity terms often depends on authors’ preference for words. We found that *digital footprints*, *children’s identity*, *online identity*, and *digital identity* were used interchangeably by authors. Together with *sharenting*, these 4 constructs were the most used terms across the articles, suggesting that they are closely related.

#### Digital Identity Creation: What and Why

We found that most of the content shared by parents was related to *social digital identity* and included sharing special events such as birthdays and family gatherings, as well as everyday activities and leisure time. In the *performative digital identity* category, posts also included content about everyday activities and leisure time but with a focus on children who were posing for a photo, with some posts contributing to the posters’ income (eg, influencers). In the *performative digital identity* category, the motives of some parents were to sell products or promote themselves and their children. The content posted appeared carefully prepared and polished. The literature on the digital identity of children frequently made reference to the concepts of safety on the internet and the rights of the child, and these 2 areas will be explored further with reference to the findings of this review.

#### Safety Risks: Digital Footprints

Although some awareness among parents of the potential risks of creating digital footprints via sharenting and the creation of their children’s digital identities was noted, there is still uncertainty about the exact impact and consequences of parental sharing behavior. One of the potential risks, digital kidnapping, was considered by some parents; however, the benefits of sharing were described as outweighing the risks of creating digital footprints and identities [9]. The perceived risks of sharenting may differ depending on the parents’ cultural background. For instance, in the study by Wagner and Gasche [61], 60% of German and Austrian mothers reported never having shared a photo of their children on the web. In an Australian study, participants refrained from posting about their children on social media as a strategy for privacy [68]. Other researchers suggest that parents who perceive web-based social networks as a source of support are highly likely to sharent [69,70].

To make an informed decision about whether to share children’s content on the web, parents need to receive information and guidance. Researchers and policy makers have started to develop new policies and guidelines for parents. Although there is a need to update existing policies to reflect the addition of online identities [71-73], the focus of many of these guidelines and policies is on children’s screen time exposure and not on children’s digital identity development or children’s right to their digital identity and footprints [71,74,75]. Therefore, we recommend more rigorous research on parents’ attitudes toward privacy and the factors influencing their sharing of children’s photos and information on the web. Findings from such studies could inform efforts and emerging policies directed at mitigating sharenting behaviors that are associated with web-related risks.

#### Children’s Rights and Privacy

The process of children’s digital identity creation most often takes place without the child’s permission or input [10,17-19,43,45,52-54,62]. No studies in this review investigated young children’s creation of their own digital identities on social networking sites. A study in this review asked children for their opinion on their parents’ sharenting behavior [59], and very few of the studies in this review (4/27, 15%) addressed the agency of the child [18,19,54,59]. When digital identities are created early for the child without the input of the child, their right to create their own digital footprint or identity is taken away, leaving them without a voice and choice [45,54,60]. Where possible, children should be involved in the development of their digital identity. Research to identify how this can be achieved and to give voice to the experiences of young children is needed to better understand this important and fast-moving area [19]. Future studies should explore the perspectives of children as key stakeholders in the creation of their digital identity [19,76].

### Strengths and Limitations

To our knowledge, this is the first scoping review to map out the literature published on the creation of digital identities among young children through social networking sites. We strove to apply rigorous methods to search and select articles and chart the data. Owing to our strict age range exclusion criteria, we did not review articles that discussed the digital identity of children aged ≥9 years on social networking sites. The use of search terms and the selected databases may not have been exhaustive, and the omission of social networking sites such as YouTube is a limitation. The search was only valid up to April 2023. In the same vein, most of the included studies were conducted in the Western world, with only 7% (2/27) of the studies conducted in Asia and none conducted in Africa or South America. The interpretation of the findings should consider this geographical bias.

### Conclusions

Digital identities on social networking sites are created when photos and information about a person are shared. The digital identities of children on social networking sites from conception to the age of 8 years are most often created by their parents (without the children’s permission). Children’s digital identities can be grouped into 2 categories: social and performative. Parents use the web environment to capture moments that matter to them while also creating positive narratives around the child’s life. The content that is shared for each type of identity and the motivation behind the creation of such identities differ. Research into young children and the digital world has focused on areas such as the effects of screen time and child development and digital safety [77-81]. We urge greater attention to the important area of how the digital identity is created, the impact of this, and how young children can be involved in important decisions that affect their lives.

## Appendix

Multimedia Appendix 1 PRISMA (Preferred Reporting Items for Systematic Reviews and Meta-Analyses) checklist and flowchart of the study selection and inclusion process.

Multimedia Appendix 2 Search strategy.

## Abbreviations

PRISMA: Preferred Reporting Items for Systematic Reviews and Meta-Analyses
PRISMA-ScR: Preferred Reporting Items for Systematic Reviews and Meta-Analyses extension for Scoping Reviews

## Data Availability

The data sets generated during and analyzed during this study are available from the corresponding author on reasonable request.
