# Supplementary figures and images for: Young Children and the Creation of a Digital Identity on Social Networking Sites: Scoping Review
Source: JMIR Pediatr Parent. 2024 Feb 21;7:e54414. doi: 10.2196/54414 (PMC10918551; doi:10.2196/54414)

## Appendix I


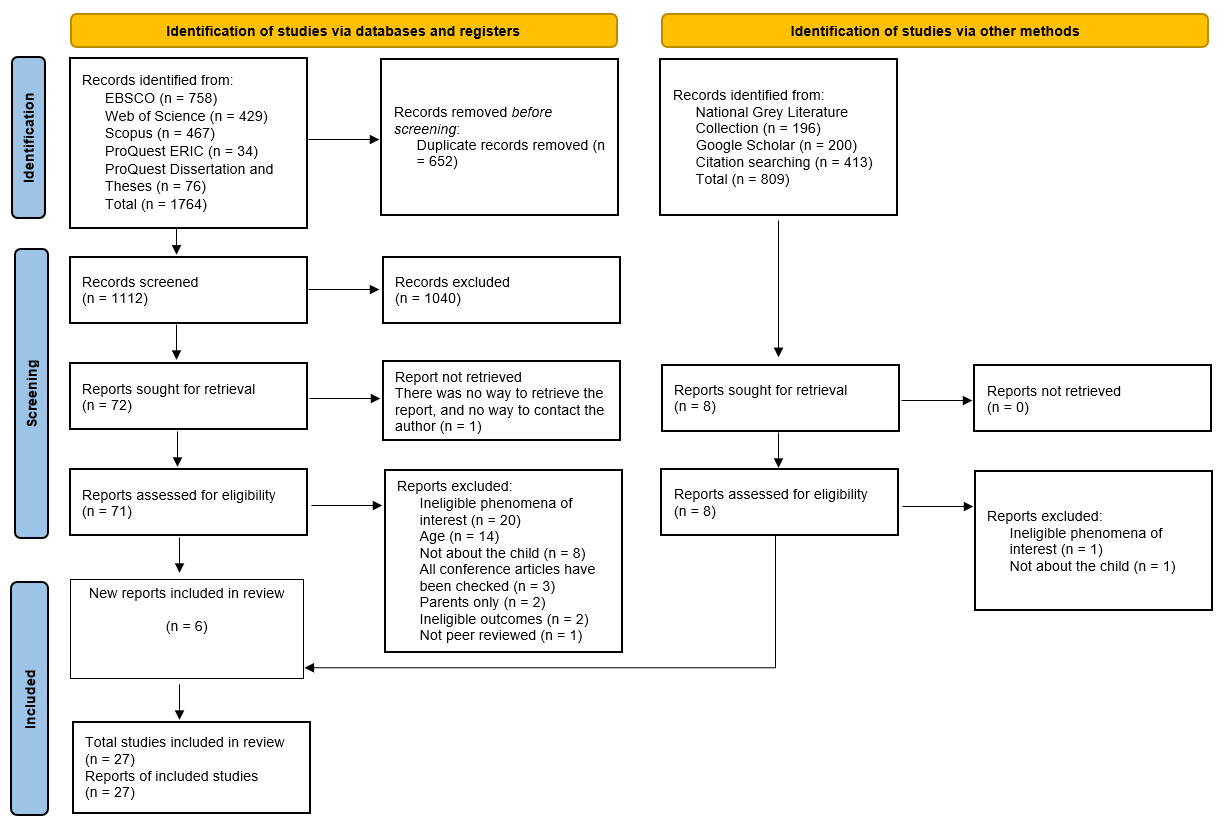
Figure 1: PRISMA Flowchart of the Study Selection and Inclusion Process

Supplement: Multimedia Appendix 1 [file pediatrics_v7i1e54414_app1.docx]
