# Supplementary material for: Young Children and the Creation of a Digital Identity on Social Networking Sites: Scoping Review
Source: JMIR Pediatr Parent. 2024 Feb 21;7:e54414. doi: 10.2196/54414 (PMC10918551; doi:10.2196/54414)
Supplement: Multimedia Appendix 2 [file pediatrics_v7i1e54414_app2.docx]

## Appendix II

### Search Strategy

Population: child OR children OR infant OR toddler OR preschooler

AND

Concept: (digital AND identity) OR "digital identity" OR (online AND profile) OR "online profile" OR (social AND presence) OR "social presence" OR sharenting

AND

Context: "social media" OR facebook OR instragram OR twitter OR snapchat OR Tumblr OR "social networking"

Limits: English and 2000 -2023.
